# The Role of Recombinant Human Cyclophilin A in the Antitumor Immune Response

**DOI:** 10.32607/20758251-2019-11-2-63-67

**Published:** 2019

**Authors:** A. A. Kalinina, Yu. Yu. Silaeva, D. B. Kazansky, L. M. Khromykh

**Affiliations:** Federal State Budgetary Institution «N.N. Blokhin National Medical Research Center of Oncology» of the Ministry of Health of the Russian Federation, Kashirskoye Sh. 24, Moscow, 115478, Russia; Federal State Budget Institution of Sciences «Institute of Gene Biology» Russian Academy of Sciences, Vavilova Str. 34/5, Moscow, 119334, Russia

**Keywords:** Cyclophilin A, pro-inflammatory factor, antitumor immune response, transgenic mice, T-cell receptor

## Abstract

Cyclophilin A (CypA) is a multifunctional protein that exhibits an isomerase activity and exists in the intracellular and secretory forms. Secretory CypA promotes regeneration of the hematopoietic and the immune systems of an organism by stimulating stem cell migration from the bone marrow. New approaches based on CypA are currently being developed for the treatment of limb ischemia, neutralization of the side effects of Cyclosporine A (CsA) therapy, etc. However, the role of CypA in the antitumor immune response is still unexplored. In this work, we used the model experimental system of lymphoma EL-4 rejection in B10.D2(R101) mice and showed that recombinant human CypA (rhCypA) stimulates the antitumor immune response via early recruitment of granulocytes to the tumor cell localization site and rapid accumulation of effector T-killers

## INTRODUCTION

Cyclophilin A (CypA) is a member of the peptidyl-prolyl isomerase family and exists in the intracellular and secretory forms. Cytosolic CypA is detected in all tissues and has multiple functions [1]. This protein takes part in signal transduction through the T-cell receptor (TCR) [1]. Being a ligand for Cyclosporine A, the protein mediates its immunosuppressive action [1].

Secretory CypA is a pro-inflammatory factor that attracts innate immunity cells (granulocytes, macrophages, and dendritic cells) to the inflammation site and mediates the pathogenesis of various diseases [1]. The protein acts as a chemoattractant for stem cells, immature granulocytes, and the progenitors of dendritic cells, T- and B-lymphocytes; and induces the migration of these cells from the bone marrow to peripheral organs [2]. In this regard, CypA takes part in regenerative processes. CypA regulates the action of other chemokines and the production of pro-inflammatory cytokines [3]. CypA was shown to induce the differentiation and maturation of dendritic cells, and to enhance antigen uptake and presentation by these cells [4]. Hence, CypA can modulate both the innate and the adaptive immunity. A vast body of experimental data suggests the application of CypA in the treatment of viral diseases and limb ischemia, for neutralizing the side effects of Cyclosporine A, etc.

However, the role of CypA in the induction and development of the antitumor immune response remains poorly understood to date. The aim of the present study was to determine the functions of CypA in the early stages of the antitumor immune response. Here, we studied the effect of recombinant human CypA (rhCypA) on the rejection of lymphoma EL-4 cells in B10.D2(R101) mice. The immunomodulatory effect of rhCypA was identified, aimed at stimulating both the innate and the adaptive immune system. As a result, accelerated *in vivo *elimination of lymphoma cells was observed under rhCypA treatment. Moreover, it was shown using the model of antitumor immune response to lymphoma EL-4 in transgenic 1D1b mice [5] that rhCypA stimulates the accumulation of tumor-specific cytotoxic T cells.

## MATERIALS AND METHODS

**Mice**

C57BL/6 (KbI-AbDb) and B10.D2(R101) (KdI-AdI-EdDb) mice were obtained from the breeding facility of the N.N. Blokhin National Medical Research Center of Oncology (Moscow, Russia). The transgenic mouse line 1D1b was generated on the genetic background of the B10.D2(R101) line in the Laboratory of Regulatory Mechanisms in Immunity of the N.N. Blokhin National Medical Research Center of Oncology [5]. These transgenic mice are characterized by expression of the β-chain of the memory-cell TCR, specific to the molecule of the major histocompatibility complex (MHC) class I H-2Kb, in T cells. Female and male mice (16–18 g) were used in the experiments. The study groups consisted of 6–8 animals. All the experimental procedures were conducted in strict compliance with the protocols approved by the Ethics Committee on Animal Experimentation of the N.N. Blokhin National Medical Research Center of Oncology.

**Production of rhCypA**

The recombinant protein was isolated from the bacterial biomass of *E. coli *BL21(DE3)Gold transformed with the recombinant plasmid pETCYPopti that contained the full-length gene of human CypA [6]. RhCypA was used as a solution in Na-K phosphate buffered saline (PBS, pH 7.3) with purity above 95% according to electrophoresis data. The endotoxin content in the rhCypA samples was ≤ 0.038 ng per 1 mg of the protein according to the LAL test.

**Immunization of mice**

B10.D2(R101) and 1D1b mice were i.p. immunized with lymphoma EL-4 (KbDb) cells at doses of 3.0 × 10^5^ and 1.0 × 106, respectively, in 500 μl PBS.

**Mode of rhCypA administration**

B10.D2(R101) mice were i.p. injected with 5 mg rhCypA/kg (100 μg/mouse) during 3 days post-immunization. The first protein injection was made 3 h post-implantation of EL-4 cells. 1D1b mice were subcutaneously dosed with 10 mg rhCypA/kg during 10 days post-immunization. Control mice received PBS as a placebo in a similar manner.

**Cell isolation**

B10.D2(R101) mice were euthanized by cervical dislocation on days 6, 9, and 12 post-immunization. The peritoneal cavities of mice were washed with 2 ml of ice-cold PBS to obtain the lavage. Splenocyte suspensions were prepared by isolating the murine spleens and homogenizing in a Potter tissue homogenizer in 3 ml of PBS. The 1D1b transgenic mice were euthanized on day 12 post-immunization; their splenocytes were isolated using a similar procedure. Erythrocytes were lysed in a lysis buffer (BD, USA). Cells were then washed in PBS and centrifuged (200 g, 5 min). Viable cells were counted in a Goryaev chamber after Trypan Blue-Eosin staining.

**Antibodies**

The following monoclonal antibodies were used for the analyses: anti-CD3ε – eFluor450 (clone 17A2) (eBioscience, USA); anti-CD8 – Pacific blue (clone 53-6.7) (BD Pharmingen, USA); anti-CD44 – APC (clone IM7) (eBioscience); anti-CD62L – APC-Cy7 (clone MEL-14) (eBioscience); anti-Vb6 – PE (clone RR4-7) (eBioscience); anti-Gr1 – APC (clone RB6-8C5) (BD Pharmingen); and anti-CD11b-PE – Cy7 (clone M1/70) (BD Pharmingen).

**Flow cytometry analysis**

The lavage and spleen cell samples (1.0–5.0 × 106) were incubated with Fc block (clone 2.4G2, BD Pharmingen, USA) for 5 min at 4°C and stained with monoclonal antibodies for 40 min at 4°C. Cells were then washed with PBS by centrifugation (200 g, 5 min), followed by analysis on a FACS CantoII flow cytometer (BD, USA) using the FACSDiva 6.0. software. Dead cells were excluded from the analyses comprising staining with propidium iodide (BD, USA). In order to characterize cell subpopulations, 0.5–1.0 × 10^6^ events were analyzed. The FlowJo 7.6. software (BD, USA) was used for further processing of the results.

**Statistical analysis**

Statistical data analysis was performed using the Student’s t-test in Excel (Microsoft, USA). The differences were considered statistically significant at *p *≤ 0.05.

## RESULTS

**Fig. 1:**
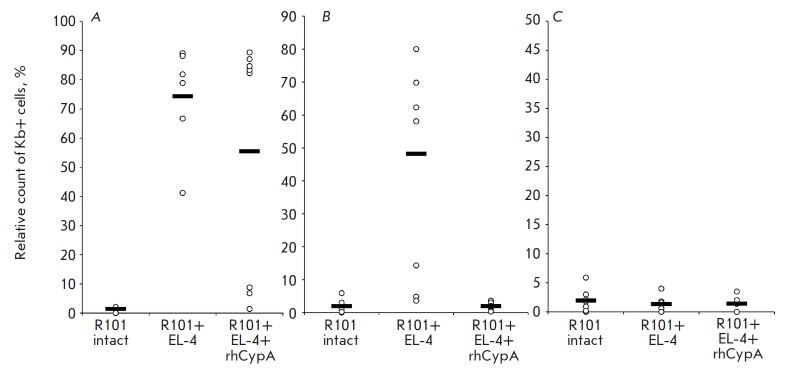
The relative count of lymphoma EL-4 cells (Kb+, %) in the peritoneal cavity of B10.D2(R101) mice on days 6 **(***A***)**, 9 **(***B***) **and 12 **(***C***) **post-immunization. Data obtained in three representative experiments are shown (M ± SD, n = 6–8). The relative count of Kb+ cells in the lavage of intact mice represents the level of unspecific binding of anti-Kb monoclonal antibodies

In this study, we used an allogenic system in which the EL-4 (KbDb) lymphoma cells were rejected in B10. D2(R101) (KdI-AdI-EdDb) mice because of the difference in a single MHC I class molecule (H2-Kb). It was shown that rhCypA administration results in complete EL-4 elimination by day 9 post-transplantation, whereas complete tumor rejection in the absence of rhCypA was observed on day 12 (*Fig. 1*).

**Fig. 2:**
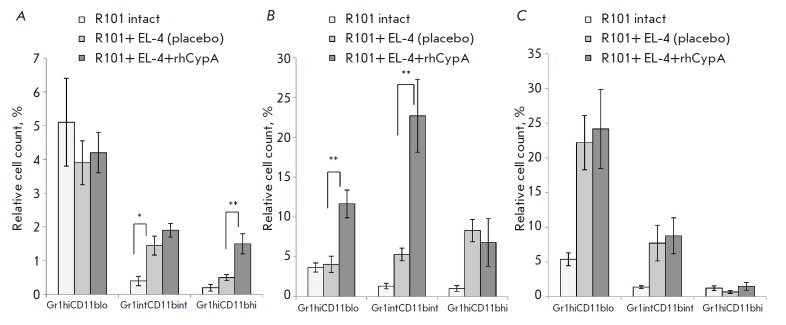
Changes in the relative count (%) of immature neutrophils (Gr1hi CD11blo), promyelocytes and myelocytes (Gr1int CD11bint), and mature granulocytes (Gr1hi CD11hi) in the peritoneal cavity of B10.D2(R101) mice on days 6 (*A*), 9 (*B*), and 12 (*C*) post- immunization with lymphoma EL-4 cells. Data obtained in three representative experiments are shown (M ± SD, n = 6–8). *p ≤ 0.05; **p ≤ 0.01

The immune response to EL-4 was accompanied by granulocyte accumulation at the tumor cell localization site. The rhCypA induced intensive accumulation of mature granulocytes in the peritoneal lavage of mice on day 6 post-immunization: the relative count of these cells was threefold higher than that in the immunized control mice (*Fig. 2A*). On day 9 post-immunization, rhCypA stimulated recruitment of immature granulocytes (Grhi CD11blo) and promyelocytes and myelocytes (Grint CD11bint) in the peritoneal cavity of dosed mice. Cell counts in these subpopulations were increased under rhCypA administration 2.5- and 4.5- fold, respectively, compared to the respective cell counts in immunized control mice (*Fig. 2B*).

**Fig. 3:**
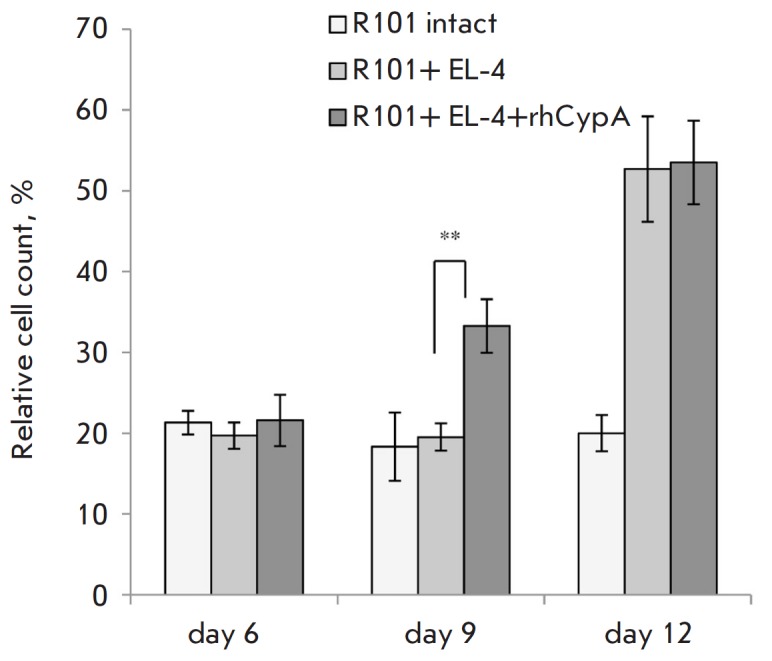
The accumulation dynamics of effector CD8+ T-lymphocytes (CD62L-CD44+) in the spleen of B10. D2(R101) mice after immunization with lymphoma EL-4 cells. Data obtained in three representative experiments are shown (M ± SD, n = 6–8). **p ≤ 0.01

Next, we evaluated the effects of rhCypA on quantitative and subpopulation changes in CD8+ T cells in tumor-bearing mice. The protein under study did not influence the dynamics of CD8+ T cell accumulation neither in the tumor localization site (as assessed by lavage analyses) nor at the systemic level (as assessed by splenocyte analyses; data are not shown). However, analyses of CD8+ T cell subsets of naive cells (CD62L+CD44-), central memory cells (CD62L+CD44+), and effectors (CD62L-CD44+) revealed that rhCypA induced enhanced accumulation (by 65% as compared to the placebo control) of effector cytotoxic T cells on day 9 post-transplantation of EL-4 tumor cells (data are not shown, *Fig. 3*). These data correlated well with the tumor rejection dynamics under rhCypA treatment (*Fig. 1*).

Therefore, it has been demonstrated that intraperitoneally injected rhCypA stimulated the antitumor immune response by inducing early recruitment of granulocytes to the tumor cell localization site and enhancing systemic accumulation of effector T-killers.

It was previously shown at our laboratory that transgenic 1D1b mice developed a significantly reduced pool of effector CD8+ T cells in response to EL-4 cells as compared to wild-type mice. Consequently, 1D1b mice could not reject this lymphoma [5, 7].

In this study, we evaluated the effects of rhCypA on the relative count of effector T cells expressing either endogenous TCR β-chains or the transgenic TCR β-chain as defined by anti-Vb6 antibody staining in 1D1b mice.

**Fig. 4:**
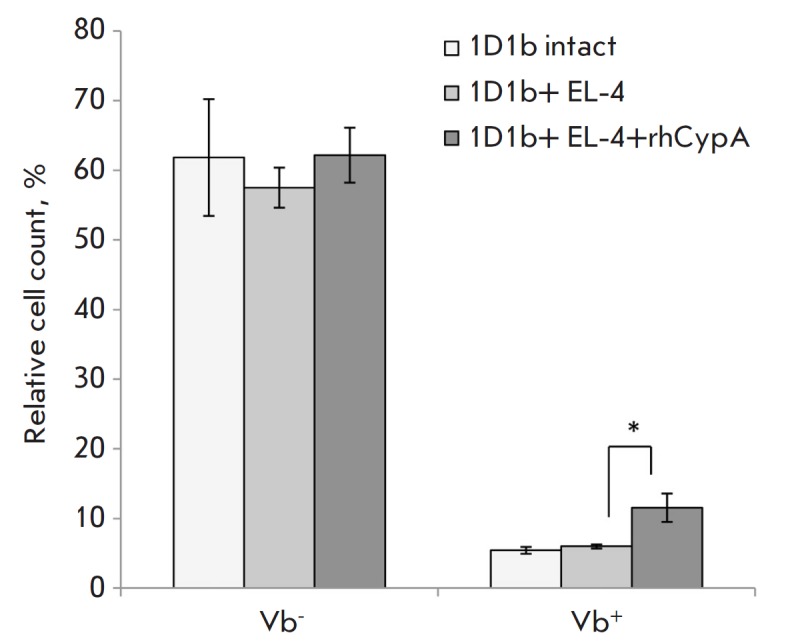
The relative count (%) of effector CD8+ T-lymphocytes (CD62L-CD44+) with the endogenous TCR β-chains (**Vb6-**) or transgenic TCR β-chain (**Vb6+**) in the spleen of 1D1b transgenic mice on day 12 post-immunization. Data obtained in three representative experiments are shown (M ± SD, n = 6–8). *p ≤ 0.05

The *in vivo *experiments showed that rhCypA had no effect on the relative count of effector CD8+ T cells with endogenous TCR β-chains in 1D1b mice immunized with EL-4 cells (*Fig. 4*). Interestingly, administration of rhCypA significantly increased (2.0- fold as compared to the placebo control) the count of effector CD8+ T cells with the transgenic TCR β-chain (*Fig. 4*).

According to these data, we have assumed that rhCypA can modulate an antitumor immune response both in mice with the native TCR repertoire (B10. D2(R101)) and in transgenic 1D1b mice with the contracted TCR repertoire by inducing the accumulation of effector CD8+ T cells.

## DISCUSSION

In this study, we evaluated the role of rhCypA in the development of the antitumor immune response to lymphoma EL-4 cells in B10.D2(R101) mice. It was shown that rhCypA stimulates granulocyte accumulation at the tumor cell localization site and systemic accumulation of effector cytotoxic T cells, which results in rapid tumor elimination. It is well-known that tissue infiltration with neutrophils is the first phase of the immune response to infections and inflammation. These cells can take up an antigen and migrate to the draining lymph nodes and the spleen, where neutrophils come in contact with the antigen-presenting cells (APCs) and lymphocytes [8] or directly function as APCs [9], thus inducing the formation of the adaptive immune response. We have previously shown that neutrophils participate in the development of the immune response to allogenic tumor cells [10]. These cells could provide co-stimulatory signals (CD80 and CD86) and create the cytokine microenvironment (interleukin 12), which are both required for differentiation of cytotoxic T cells [10, 11]. It was shown in the present study that local processes in the peritoneal cavity taking place under rhCypA treatment correlate with the systemic immune response.

In 1D1b mice, the expression of the transgenic TCR β-chain in T cells both contracted the TCR repertoire and reduced the count of activated T cells [5]. The immune response to EL-4 in 1D1b transgenic mice was insufficient for complete tumor elimination and drove the immunoediting of EL-4 cells via the selection of less immunogenic tumor cell clones that killed transgenic mice within 60 days [7]. It was shown using this experimental model that rhCypA significantly stimulates the accumulation of tumor-specific cytotoxic T cells at the early phases of the immune response to lymphoma EL-4. These data allow one to assume that rhCypA can modulate the antitumor immune response both in mice with the native TCR repertoire and in those with the contracted TCR repertoire by inducing the accumulation of effector T-killers.

Hence, we have demonstrated that rhCypA has an immunostimulating effect, as it facilitates the development of the antitumor immune response by stimulating both innate and adaptive immunity.

## Abbreviations

CypA: Cyclophilin A
rhCypA: recombinant human CypA
TCR: T-cell receptor
MHC: major histocompatibility complex
PBS: phosphate buffered saline
i.p.: intraperitoneal injection
APCs: antigen-presenting cells
CD: cluster of differentiation
